# Analysis of risk factors and application of risk management strategies in hemodialysis patients complicated with heart failure

**DOI:** 10.3389/fcvm.2025.1600223

**Published:** 2025-06-16

**Authors:** Guiren Hou, Sai Chen

**Affiliations:** Emergency and First Aid Center, Changsha Fourth Hospital, Changsha China

**Keywords:** hemodialysis, heart failure, risk factors, risk management strategies, prognosis

## Abstract

**Background:**

Heart failure (HF) is a common and serious complication in maintenance hemodialysis (MHD) patients, significantly affecting their prognosis and quality of life. This study aims to identify risk factors for HF and evaluate targeted, risk-based nursing interventions.

**Methods:**

A total of 170 MHD patients admitted between January 2022 and January 2024 were divided into HF (*n* = 80) and non-HF groups (*n* = 90) based on the presence or absence of HF. Risk factors were analyzed using univariate and multivariate logistic regression. Subsequently, 80 HF patients were randomized to standard group (*n* = 40) or study group receiving targeted risk management strategies (*n* = 40). The intervention lasted 8 weeks and included comprehensive nursing measures based on individualized risk profiles. Outcome measures included Self-Rating Anxiety Scale (SAS), Self-Rating Depression Scale (SDS), complications, quality of life (SF-36), and nursing satisfaction.

**Results:**

Univariate analysis identified age (OR = 1.076), dialysis duration (OR = 1.054), hypertension (OR = 3.391), diabetes (OR = 2.874), coronary heart disease (OR = 3.115), smoking history (OR = 1.976), HbA1c (OR = 18.675), and C-reactive protein (CRP; OR = 1.466) as risk factors for HF in MHD patients (*P* < 0.05). Multivariate logistic regression analysis confirmed age (OR = 1.079), HbA1c (OR = 20.371), and CRP (OR = 1.542) as independent risk factors. After management, compared with the standard group, the study group showed significant reductions in SAS and SDS scores and complication incidence (*P* < 0.05). The quality of life and nursing satisfaction significantly improved in the study group (*P* < 0.05).

**Conclusion:**

Age, hyperglycemia, inflammation, and comorbidities (hypertension, diabetes, coronary heart disease) independently increase HF risk in MHD patients. Targeted risk management reduces psychological distress, complications, and enhances care outcomes.

## 1 Introduction

The global burden of chronic kidney disease (CKD) continues to rise, paralleled by an expanding population of patients progressing to end-stage renal disease (ESRD). Hemodialysis remains an indispensable life-sustaining therapy for these individuals, effectively alleviating uremic symptoms and improving quality of life (1). However, the hemodialysis procedure itself imposes significant physiological strain due to rapid fluid shifts, abrupt electrolyte fluctuations, and drastic hemodynamic changes during extracorporeal circulation. These acute perturbations induce systemic stress responses that aggravate cardiac workload, rendering heart failure (HF) a common and life-threatening complication in this vulnerable cohort (2, 3). Beyond worsening clinical outcomes and diminishing quality of life, HF significantly increases mortality risk through a series of pathophysiological changes, bringing great challenges to both patients and the medical system (4).

As a clinical syndrome, HF arises from multifactorial etiologies involving structural cardiac remodeling, functional abnormalities, and multi-system interactions (5). For hemodialysis patients, they frequently harbors comorbid conditions such as hypertension, diabetes and coronary heart disease, which are chronic pathologies that promote myocardial hypertrophy, arteriosclerosis and endothelial dysfunction in the long run, thus further increasing the risk of HF (6–8). In addition, hemodialysis-associated hemodynamic instability, chronic fluid overload, and electrolyte disturbance synergistically drive HF to varying degrees (9, 10). Consequently, how to early identify and effectively intervene these modifiable risk factors during hemodialysis has become an important topic to mitigate HF progression.

Despite advances in personalized medicine and the growing emphasis on comprehensive risk stratification, current clinical practices lack standardized protocols for holistic risk assessment and management in hemodialysis populations (11). Existing studies predominantly focus on isolated biomarkers or short-term efficacy, often neglecting the dynamic interactions between multimorbidity profiles, hemodialysis parameters, and longitudinal cardiovascular sequelae. Furthermore, fragmented research approaches have hindered the development of unified clinical frameworks for risk mitigation. In this context, the integration of modern diagnostic modalities, including multidimensional biomarker profiling, big data analysis, and precision medicine principles, holds transformative potential for constructing robust risk prediction models and tailored intervention strategies for HF in hemodialysis patients.

Therefore, this study seeks to address these gaps by establishing a structured, risk-oriented care model for HF prevention in patients undergoing maintenance hemodialysis (MHD). Through systematic analysis of clinical, biochemical, and hemodialysis-related parameters, we intend to identify key predictors of HF development, implement individualized interventions guided by risk stratification, and assess their impact on psychological status, complication rates, quality of life, and care satisfaction. Ultimately, this initiative aims to facilitate a shift from empirical to scientific and refined risk management in hemodialysis care, providing new strategies for reducing HF-related hospitalization and mortality.

## 2 Data and methods

### 2.1 Study design

This study adopted a two-phase design. First, a retrospective case-control study was conducted to identify potential clinical risk factors for HF among patients undergoing MHD. Based on the presence or absence of HF, patients were divided into HF and non-HF groups for univariate and multivariate logistic regression analysis. Following this, a prospective, controlled intervention trial was implemented in a subset of patients diagnosed with HF to assess the efficacy of a targeted, risk-stratified nursing management strategy. This sequential approach was considered appropriate as it enabled the identification of high-risk factors through observational data while also allowing a structured evaluation of a tailored care model in clinical practice.

### 2.2 Sample size calculation

The risk factors for HF in MHD patients were identified using logistic regression analysis. The sample size for this case-control study was calculated using the Hsieh formula: $n=\frac{{\left(Z_{1-\alpha /2}+Z_{1-\beta }\right)}^{2}∙P(1-P)(r+1)}{r∙{\left(P_{1}-P_{0}\right)}^{2}}$, where $Z_{1-\alpha /2}=1.96$ (two-sided *α* = 0.05), $Z_{1-\beta }\;=0.84$ (*β* = 0.2, 80% power), *P*_0_ = exposed cases (such as hypertension) in the control group, *P*_1_ = exposed cases in the case group, and r = control-to-case ratio. According to literature report (12), the hypertension exposure rate P_0_ in the HF group was about 60%, and the hypertension exposure rate P_1_ in the non-HF group was about 40%. $P=\frac{P_{1}+rP_{0}}{1+r}=\frac{0.6+1.125\times 0.4}{1+1.125}\approx 0.48$. By substituting the above formula, n was equal to about 70/group, and the total sample size needed to be about 140 cases. Considering the shedding rate of 10%, 165 cases or more were required to accommodate multivariate analyses.

For the intervention trial comparing the nursing effects between the two groups [such as Self-Rating Anxiety Scale (SAS) score reduction], the sample size was calculated using the t test formula for two independent samples: $n=\frac{2{\left(Z_{\alpha /2}+Z_{1-\beta }\right)}^{2}∙SD^{2}}{\Delta^{2}}$. Thereinto, Δ=5 (expected score variance), SD = 10 (score standard deviation) $Z_{\alpha /2}\;=1.96$, $Z_{\beta }\;=0.84$, and n was yielded to be approximately 32 cases/group. Considering a 10% dropout rate, it was necessary to recruit 40 cases per group, over 80 cases in total, to meet the requirements for multivariate analyses. The overall sample size was designed to cover three potential risk factors and satisfy the rule of “20 events per variable” for logistic regression (i.e., 60 events). The requirement of 80 cases for intervention trial would be achieved by randomly assigning 80 cases from the HF group into the standard group and the study group (40 cases each). Therefore, a total sample size of 170 cases were included in this study to cover the requirements for both case-control study and intervention trial.

### 2.3 General data

A total of 170 MHD patients hospitalized in our hospital from January 2022 to January 2024 were collected and sorted out, including 119 males and 51 females, aged 31–75 years old. According to the presence or absence of HF, they were divided into HF group (*n* = 80) and non-HF group (*n* = 90). The diagnosis of HF was based on the 2021 European Society of Cardiology (ESC) Guidelines for the Diagnosis and Treatment of Acute and Chronic Heart Failure (13), incorporating both clinical symptoms (e.g., dyspnea, fatigue, edema) and objective evidence of cardiac dysfunction. All patients underwent transthoracic echocardiography by experienced sonographers within 48 h of admission. The left ventricular ejection fraction (LVEF) was measured using the biplane Simpson's method, with HF classified as: HFrEF (HF with reduced ejection fraction): LVEF < 40%; HFmrEF (mildly reduced): LVEF 40%–49%; HFpEF (preserved): LVEF ≥ 50% with structural heart disease or diastolic dysfunction. General data of the two groups of patients were collected and recorded for analysis.

### 2.4 Inclusion and exclusion criteria

Inclusion Criteria: ① Patients were aged 18 years or older who had been undergoing regular MHD for more than three months. ② Patients were in good cardiopulmonary health without severe acute or chronic diseases, and were capable of undergoing the study-related examinations and treatments. ③ Patients had not undergone major surgeries or experienced acute complications within the three months prior to enrollment, and their conditions were stable. ④ Patients demonstrated high compliance by following medical advice and regularly attending dialysis sessions and related examinations. ⑤ Patients were able to understand the study objectives, had signed the informed consent form, and were willing to cooperate with follow-up visits and long-term observation.

Exclusion criteria: ① Patients were excluded if they had severe liver diseases (e.g., cirrhosis or liver failure), significant systemic infections, active tuberculosis, malignant tumors, connective tissue diseases, or other major illnesses. ② Patients with congenital kidney diseases, congenital heart defects, or other severe congenital structural abnormalities were excluded. ③ Patients who had a documented history of severe cardiac diseases were excluded, including those with primary/secondary cardiomyopathy, valvular heart disease, myocarditis, or pericardial diseases. ④ Patients were excluded if they had severe mental disorders or cognitive impairments that prevented their cooperation with study assessments or treatments. ⑤ Patients whose clinical records or examination data were incomplete, thereby precluding effective analysis, were excluded.

### 2.5 Intervention strategy

The 80 HMD patients with HF were randomly assigned to the standard group (*n* = 40) and the study group (*n* = 40). The standard group received conventional care using a comprehensive management protocol for haemodialysis patients with concomitant HF. The interventions included: (1) Continuous monitoring of vital signs (blood pressure, respiratory rate, pulse, and heart rhythm); (2) Administration of supplemental oxygen therapy to optimise tissue oxygenation; (3) Instruction on effective coughing techniques to facilitate airway clearance; (4) Strict fluid volume management with timely correction of hydro-electrolyte imbalances; (5) Implementation of metabolic support therapies; (6) Assistance with positional adjustments, predominantly adopting an upright posture with lower limb dependency; (7) Environmental regulation to maintain appropriate ambient temperature (22–24℃) and humidity (50%–60%); (7) Provision of individualised dietary counselling to ensure nutritional adequacy for both dialysis requirements and cardiac functional status.

The study group received risk-stratified management interventions in addition to the standard care protocol. These interventions comprised four key components: (1) System enhancement: A standardized nursing protocol and an accountability frameworks were established. Regular competency-based training sessions were conducted for nursing staff, enhancing emergency response capabilities and fluid management expertise. Individualized care plans were developed based on risk profiles, with targeted interventions such as intensified glycemic control for diabetic patients and optimized blood pressure monitoring regimens for hypertensive cases. (2) Risk stratification: High-risk patients were identified through comprehensive admission assessments and systematic follow-up evaluations, with dedicated risk profiles documented. Enhanced hemodynamic monitoring systems incorporating alert thresholds were implemented to enable early detection and management of clinical deteriorations. Strict pharmacological supervision and fluid balance protocols were enforced to mitigate complication risks. (3) Environmental modification: The dialysis unit environment was optimized through precise temperature (22–24°C) and humidity (50%–60%) regulation, complemented by stringent infection control measures. Dedicated cardiac care zones were established for HF patients. (4) Quality control: A multidimensional quality monitoring system was implemented, with quarterly audits of critical care domains including: patient education effectiveness, vital sign documentation accuracy, compliance with nursing protocols, and patient satisfaction metrics. Corrective actions were promptly initiated for identified deficiencies to optimize procedural safety and clinical outcomes.

The interventions were delivered two to three times per week, integrated into both dialysis sessions and peri-dialysis periods (before and after dialysis), over a total duration of eight weeks. Interventions were designed and administered by a dedicated nursing team following a standardized protocol. While the nurses delivering the interventions were aware of group allocation due to the nature of the care, outcome assessments were performed by independent evaluators blinded to group assignment to minimize bias. It is important to note that all participants continued their routine pharmacological treatments throughout the study period, including antihypertensive, antidiabetic, and cardioprotective medications, as prescribed by their attending physicians. The present study did not introduce any modifications to drug regimens. The core of the interventions was based on risk factor-oriented nursing management strategies, including individualized care planning, psychological support, hemodynamic monitoring, patient education, and environmental optimization. These measures were applied in addition to, but independently of, existing pharmacological treatments to evaluate the independent effect of structured, non-pharmacological risk management on patient outcomes.

### 2.6 Observational indices

Outcome indicators including SAS, Self-Rating Depression Scale (SDS), MOS 36-Item Short-Form Health Survey (SF-36) scores, complication incidence, and nursing satisfaction were assessed at baseline and at the end of the 8-week intervention period.

The psychological states of patients before and after management were compared between the two groups using SAS (14) and SDS (15, 16). Both tools employ standardized 0–100 scoring systems, where higher scores indicate greater severity of anxiety and depression.

The quality of life was longitudinally assessed using SF-36, with domains of physiological function, social function, emotional function, mental health, and general health selected (17, 18). Each domain is standardized to a 0–100 scale. A higher score indicates superior functional status and well-being across physical, emotional, and social dimensions. Conversely, a lower score reflects poorer quality of life, with patients likely to experience more discomforts, reduced capacity for daily activities, and poorer physical and mental health.

Complications were continuously monitored and recorded throughout the 8-week intervention period. Data on complications were collected from patients' electronic medical records and daily nursing logs, including events such as pulmonary edema, arrhythmia, hypotension, electrolyte imbalance, and dialysis-related infection. All diagnoses were confirmed by attending physicians based on established clinical criteria. To minimize potential bias, all complication data were reviewed and classified by independent evaluators who were blinded to group allocation.

The nursing satisfaction was evaluated by a self-made 25-item questionnaire, employing a 4-point Likert scale (1 = very dissatisfied, 2 = dissatisfied, 3 = satisfied, and 4 = very satisfied). The scale is scored out of 100 points, with scores above 90 considered as very satisfied, between 70 and 90 as satisfied, and below 70 as dissatisfied. Overall satisfaction was defined as the sum of “very satisfied” and “satisfied” responses. This scoring system aims to comprehensively evaluate patients' overall feelings about nursing services, covering multiple dimensions such as nursing quality, service attitude, communication effect and treatment effect.

### 2.7 Statistical analysis

SPSS 26.0 software was used for data analysis. After assessment for normality, data conforming to a normal distribution were presented as mean ± standard deviation ($\bar{x}\pm s$). For data that did not conform to the normal distribution, the median and quartile [M (Q25, Q75)] were adopted. Independent sample t test was used for the measurement data between two groups. Categorical data were expressed as *n* (%), and their differences were analyzed by *χ*^2^ test. Univariate and multivariate logistic regression analyses were applied to identify independent risk factors for HF in MHD patients, with statistical significance set at *P* < 0.05.

## 3 Results

### 3.1 Univariate analysis of influences on Hf in hemodialysis patients

Among CKD patients on hemodialysis, those with HF were significantly older (OR = 1.076, 95% CI: 1.038–1.115) and had longer dialysis duration (OR = 1.054, 95% CI: 1.002–1.109), as well as a higher prevalence of hypertension (OR = 3.391, 95% CI: 1.789–6.430), diabetes (OR = 2.874, 95% CI: 1.520–5.432), coronary heart disease (OR = 3.115, 95% CI: 1.659–5.849), and smoking history (OR = 1.976, 95% CI: 1.071–3.646). They also exhibited higher levels of glycosylated hemoglobin (HbA1c; OR = 18.675, 95% CI: 4.531–76.977) and CRP (OR = 1.466, 95% CI: 1.255–1.712) compared to those without HF (*P* < 0.05; as seen in Tables 1, 2).

**Table 1:** Univariate analysis of influencing factors for HF in hemodialysis [($\bar{x}\pm s$), M (Q25, Q75), *n* (%)].

Factor		HF group (*n* = 80)	Non-HF group (*n* = 90)	$x^{2}t/Z$	*P*
Gender	Male	58 (72.50%)	61 (67.78%)	0.450	0.502
	Female	22 (27.50%)	29 (32.22%)		
Age (years)		62 (58,68.5)	57 (48,65)	4.942	<0.001
Body mass index (kg/m^2^)		25 (23,26)	25 (23,26)	0.338	0.735
Dialysis duration (months)		24.03 ± 5.65	22.09 ± 6.48	2.077	0.039
Hypertension history	Yes	57 (72.25%)	38 (42.22%)	14.475	<0.001
	No	23 (28.75%)	52 (57.78%)		
Diabetes history	Yes	42 (52.50%)	25 (27.78%)	10.840	0.001
	No	38 (47.50%)	65 (72.22%)		
Coronary heart disease history	Yes	54 (67.50%)	36 (40.00%)	12.856	<0.001
	No	26 (32.50%)	54 (60.00%)		
Smoking history	Yes	49 (61.25%)	40 (44.44%)	4.795	0.029
	No	31 (38.75%)	50 (55.56%)		
Drinking history	Yes	43 (53.75%)	45 (50.00%)	0.239	0.625
	No	37 (46.25%)	45 (50.00%)		
Hemoglobin (g/L)		87.13 ± 20.09	87.84 ± 17.05	0.252	0.801
Albumin (g/L)		24.78 ± 4.94	24.37 ± 4.80	0.555	0.580
Heart rate (times/min)		83 (80.25,87)	85 (82,88)	0.940	0.347
HbA1c (%)		5.73 (5.52,5.92)	5.57 (5.43,5.72)	4.025	<0.001
Triglyceride (mmol/L)		1.48 (1.23,1.69)	1.36 (1.19,1.58)	1.435	0.151
Total cholesterol (mmol/L)		3.93 (3.54,4.26)	3.85 (3.54,4.38)	0.378	0.706
Low density lipoprotein (mmol/L)		2.21 ± 0.35	2.16 ± 0.28	1.051	0.295
Troponin (ug/L)		0.036 (0.034,0.038)	0.036 (0.034,0.038)	0.598	0.550
Creatine kinase MB isoenzyme (ug/L)		1.54 ± 0.54	1.52 ± 0.47	0.349	0.727
Procalcitonin (mg/ml)		0.27 (0.25,0.29)	0.27 (0.25,0.29)	0.210	0.833
CRP (mg/ml)		9.50 (7.94,12.63)	7.61 (6.70,9.35)	5.403	<0.001
Interleukin 6 (mg/ml)		8.77 ± 2.12	9.38 ± 2.61	1.665	0.098

**Table 2:** Univariate logistic analysis of risk factors for HF associated with hemodialysis.

Factor	B	S.E	Wald	Sig	Exp (B)	95% CI
Gender	−0.226	0.337	0.449	0.503	0.798	0.412–1.544
Age	0.073	0.018	16.204	<0.001	1.076	1.038–1.115
Body mass index	0.029	0.088	0.110	0.740	1.030	0.866–1.224
Dialysis duration	0.053	0.026	4.154	0.042	1.054	1.002–1.109
Hypertension history	1.211	0.326	13.994	<0.001	3.391	1.789–6.430
Diabetes history	1.056	0.325	10.561	0.001	2.874	1.520–5.432
Coronary heart disease history	1.136	0.321	12.503	<0.001	3.115	1.659–5.849
Smoking history	0.681	0.313	4.748	0.029	1.976	1.071–3.646
Drinking history	−0.150	0.308	0.238	0.625	0.860	0.471–1.573
Hemoglobin	−0.002	0.008	0.064	0.800	0.998	0.982–1.014
Albumin	0.018	0.032	0.311	0.577	1.018	0.956–1.083
Heart rate	−0.015	0.032	0.213	0.644	0.985	0.925–1.049
HbA1c	2.927	0.723	16.409	<0.001	18.675	4.531–76.977
Triglyceride	0.667	0.529	1.586	0.208	1.948	0.690–5.498
Total cholesterol	0.082	0.287	0.081	0.777	1.085	0.618–1.906
Low density lipoprotein	0.523	0.498	1.104	0.293	1.687	0.636–4.475
Troponin	−17.662	53.195	0.109	0.741	<0.001	0.000–7.326E+37
Creatine kinase MB isoenzyme	0.107	0.306	0.123	0.726	1.113	0.611–2.029
Procalcitonin	2.313	4.815	0.231	0.631	10.109	0.002–126859.297
CRP	0.382	0.079	23.215	<0.001	1.466	1.255–1.712
Interleukin 6	−0.108	0.066	2.717	0.099	0.897	0.789–1.021

### 3.2 Multivariate analysis of factors affecting Hf in hemodialysis patients

The factors with statistical significance among the above single factors were assigned and incorporated into the multivariate model, as detailed in Table 3. Multivariate logistic regression analysis showed that age (OR = 1.079, 95% CI: 1.032–1.129), HbA1c (OR = 20.371, 95% CI: 3.350–123.864), and CRP (OR = 1.542, 95% CI: 1.265–1.879) were independent risk factors for HF in hemodialysis, as shown in Table 4.

**Table 3:** Assignment table.

Variable	Assignment
Age	Continuous variable
Dialysis duration	Continuous variable
Hypertension history	No = 0, Yes = 1
Diabetes history	No = 0, Yes = 1
Coronary heart disease history	No = 0, Yes = 1
Smoking history	No = 0, Yes = 1
HbA1c	Continuous variable
CRP	Continuous variable

**Table 4:** Multivariate logistic regression analysis of risk factors for HF associated with hemodialysis.

Factor	B	S.E	Wald	Sig	Exp (B)	95% CI
Constant	−27.137	5.776	22.077	<0.001	<0.001	–
Age	0.076	0.023	11.089	0.001	1.079	1.032–1.129
Dialysis duration	0.020	0.036	0.303	0.582	1.020	0.950–1.095
Hypertension history	0.855	0.493	3.008	0.083	2.352	0.895–6.184
Diabetes history	0.056	0.524	0.012	0.914	1.058	0.379–2.955
Coronary heart disease history	0.600	0.538	1.244	0.265	1.823	0.635–5.234
Smoking history	0.497	0.433	1.319	0.251	1.643	0.704–3.837
HbA1c	3.014	0.921	10.711	0.001	20.371	3.350–123.864
CRP	0.433	0.101	18.412	<0.001	1.542	1.265–1.879

### 3.3 Psychological states before and after risk management between two groups

Before management, there was no significant difference in SAS or SDS scores between the standard and study groups (*P* > 0.05). After management, SAS and SDS scores in the study group were significantly lower than those in the standard group, and the differences were statistically significant (*P* < 0.05), as displayed in Table 5. Additionally, it was observed that the reduction in SAS and SDS scores was accompanied by categorical downgrades in symptom severity classification for a subset of patients, suggesting not only statistical significance but also clinical improvement.

**Table 5:** Psychological states before and after risk management between standard and study groups ($\bar{x}\pm s$).

Group	SAS		SDS	
	Pre-management	Post-management	Pre-management	Post-management
Standard group (*n* = 40)	55.93 ± 8.99	46.70 ± 5.16*	54.38 ± 7.61	45.80 ± 4.35*
Study group (*n* = 40)	54.20 ± 7.11	40.35 ± 3.89*	57.20 ± 7.55	41.60 ± 5.48*
*t*	0.952	6.217	1.667	3.802
*P*	0.344	<0.001	0.099	<0.001

* *P* < 0.05.

Compared with pre-management.

### 3.4 Comparison of quality of life after risk management

Baseline assessments confirmed no significant differences in physiological function, social function, emotional function, general health, or mental health between the standard and study groups (*P* > 0.05). After management, the scores of physiological function, social function, emotional function, general health, and mental health in the study group were significantly higher than those in the standard group, with statistically significant differences (*P* < 0.05), as presented in Table 6.

**Table 6:** Effects of risk management on quality of life in standard and study groups ($\bar{x}\pm s$).

Group	Quality of life				
	Physiological function	Social function	Emotional function	Mental health	General health
Standard group (*n* = 40)	72.78 ± 5.41	72.40 ± 5.03	70.73 ± 5.51	73.30 ± 5.52	75.65 ± 5.54
Study group (*n* = 40)	84.48 ± 5.51	82.43 ± 4.96	82.68 ± 5.52	86.28 ± 5.84	84.53 ± 4.85
*t*	9.576	8.977	9.685	7.626	10.212
*P*	<0.001	<0.001	<0.001	<0.001	<0.001

### 3.5 Comparison of complications after risk management

The overall incidence of complications (hypotension, arrhythmia, pulmonary edema, electrolyte imbalance and dialysis-related infection) in the study group was 10.00% (4/40), which was significantly lower than that in the standard group (27.50%, 11/40), with statistical significance (*P* < 0.05, Table 7). During the entire observation and intervention period, no deaths occurred in either group. All enrolled patients completed the follow-up and assessment procedures. These complications encompassing hypotension, arrhythmia, pulmonary edema, electrolyte imbalance, and dialysis-related infection were effectively managed with appropriate medical interventions, without serious adverse outcomes reported.

**Table 7:** Complications in standard and study groups after risk management [*n* (%)].

Group	Hypotension	Arrhythmia	Pulmonary edema	Electrolyte imbalance	Dialysis-associated infection	Overall
Standard group (*n* = 40)	3 (7.50%)	2 (5.00%)	1 (2.50%)	2 (5.00%)	3 (7.50%)	11 (27.50%)
Study group (*n* = 40)	1 (2.50%)	0 (0.00%)	0 (0.00%)	1 (2.50%)	2 (5.00%)	4 (10.00%)
$x^{2}$						4.021
*P*						0.045

### 3.6 Comparison of nursing satisfaction after risk management

Post-management analysis demonstrated that the nursing satisfaction of the study group was 95.00% (38/40), significantly higher than that of the standard group (77.50%, 31/40), with statistical significance (*P* < 0.05), as shown in Table 8.

**Table 8:** Comparison of nursing satisfaction between study and standard groups after risk management [*n* (%)].

Group	Very satisfied	Satisfied	Dissatisfied	Overall satisfaction
Standard group (*n* = 40)	17 (42.50%)	14 (35.00%)	9 (22.50%)	31 (77.50%)
Study group (*n* = 40)	25 (62.50%)	13 (32.50%)	2 (5.00%)	38 (95.00%)
$x^{2}$				5.165
*P*				0.023

## 4 Discussion

Hemodialysis serves as a critical life-sustaining intervention for end-stage CKD, effectively substituting renal function through metabolic waste clearance and fluid homeostasis. Paradoxically, this therapeutic modality exacerbates cardiovascular morbidity, particularly HF (19), which is a prevalent complication with much higher incidence in hemodialysis patients compared to the general population (20). HF not only aggravates the underlying condition of dialysis patients, but also significantly compromises their long-term survival outcomes (21–23). This hemodialysis-associated cardiomyopathy arises through multifactorial pathogenesis, wherein fluid shifts from intermittent dialysis, cardiorenal crosstalk, chronic inflammation, and metabolic disorders synergistically drive myocardial decompensation. In view of the above factors, HF occurrence in hemodialysis patients often brings multiple challenges, elevating hospitalization frequency and mortality risk. Furthermore, this pathophysiological convergence correlates with marked deterioration in quality of life, establishing HF as both a mortality accelerator and life experience disruptor in end-stage CKD (24–26).

While advancements in dialysis technology have substantially prolonged patient survival, HF persists as an important determinant of long-term mortality in this population, necessitating refined mechanistic understanding and targeted management strategies. Our univariate analysis of hemodialysis cohorts revealed eight clinically significant HF correlates, including advanced age, extended dialysis duration, hypertension, diabetes, coronary heart disease, smoking history, higher HbA1c, and elevated CRP, collectively implicating senescence, underlying diseases, inflammatory responses, and metabolic disorders as central drivers of HF pathogenesis, which is consistent with previous findings (27, 28). The age-associated risk escalation reflects the compounding effects of cardiac senescence, manifested as reduced ventricular compliance, and dialysis-induced hemodynamic stress, which synergistically impair myocardial reserve (29). Similarly, diabetes, quantified by HbA1c, directly promotes myocardial fibrosis via advanced glycation end-product deposition (30), while CRP elevation signifies systemic inflammatory response that accelerates endothelial dysfunction and ventricular remodeling (31). Further multivariate logistic regression analysis showed that age, HbA1c, and CRP were independent risk factors for HF associated with hemodialysis, suggesting that these factors have important predictive value for HF occurrence. These findings underscore the clinical imperative for preemptive biomarker monitoring (e.g., quarterly HbA1c/CRP tracking) and stratified interventions, such as intensified volume management in elderly patients or anti-inflammatory protocols for those with persistent CRP elevation, to disrupt the HF trajectory.

Our implementation of a risk-stratified nursing management protocol, incorporating dynamic risk monitoring, biomarker-guided interventions, and personalized care pathways, yielded multidimensional improvements in hemodialysis outcomes. Post-intervention psychometric assessments revealed substantial reductions in SAS and SDS scores in the study group compared to the standard group, suggesting effectively alleviated anxiety and depression symptoms. These mental health gains paralleled significant enhancements across SF-36 domains: physiological function, social function, emotional function, general health, and mental health, indicating that this intervention can significantly improve the overall health status of MHD patients. The protocol's efficacy stemmed from its quadruple framework: Predictive risk profiling: Serial monitoring of HF drivers such as patients' age, underlying diseases, HbA1c, and CRP, enabled preemptive intervention measures, reducing HF admissions. Personalized care intervention: Personalized care plans were developed for patients with different risk factors. For example, diabetic subcohorts received strengthened blood glucose control, hypertensive patients underwent strengthened blood pressure monitoring and management, and high CRP patients were given anti-inflammatory regimens. Psychosocial integration: Structured psychiatric support protocols including psychological counseling and care of nurses effectively alleviated anxiety and depression due to long-term treatment and decreased quality of life, addressing the well-documented mind-body nexus in dialysis outcomes (32, 33). Health education and complication prevention: Health education was also provided for patients and their families to improve their awareness of HF and dialysis-related complications, thereby promoting patients' active participation in self-management and reducing the incidence of complications. This multimodal approach achieved a reduction in complication rate alongside exceptional patient satisfaction. The satisfaction gap primarily reflects a high degree of patient acceptance of this risk-stratified care.

Our findings carry important guiding significance for clinical nursing practice. First, the identification of advanced age, preexisting cardiometabolic comorbidities, and elevated HbA1c and CRP as risk factors for HF mandates protocolized risk stratification in hemodialysis units. In clinical practice, nurses should carry out regular assessment of these high risk factors paired with preemptive measures to reduce the risk of HF. Secondly, our risk-adaptive nursing model demonstrates synergistic efficacy in improving physical and mental health, reducing the incidence of complications, and enhancing the overall quality of life through comprehensive evaluation and personalized nursing intervention. Equally crucial, the increase in nursing satisfaction establishes patient-reported experience as an important quality indicator. By optimizing the way of nursing and improving nursing satisfaction, patients' compliance can be enhanced, thus achieving better treatment results. Therefore, in the future clinical nursing, we should strengthen the application of risk management, and pay attention to psychological support and health education, so as to improve the treatment effect and quality of life of patients.

Methodologically, a key feature of our study is that all clinical improvements were achieved without altering patients' medication regimens. This allowed us to evaluate the real-world impact of structured nursing strategies independently from pharmacological effects. While pharmacotherapy remains essential, our findings demonstrated that risk factor-driven nursing interventions, when designed and implemented systematically, could significantly improve physical and mental outcomes, reduce complications, and enhance satisfaction in hemodialysis patients with HF. This evidence provides an important supplement to existing drug-centered approaches and aligns with current trends in integrated care.

In summary, this study delineates age, cardiometabolic comorbidities, HbA1c, and CRP as key influencing factors of HF in hemodialysis populations. Moreover, this risk-adaptive nursing intervention establishes a replicable framework for mitigating the dual burden of physical and mental morbidity in hemodialysis-associated HF. By synergizing biomarker surveillance with personalized care pathways, we demonstrate that precision nursing can disrupt the downward spiral of dialysis-related complications while elevating patient-centered outcomes. This provides a powerful theoretical basis and practical guidance for the nursing intervention of hemodialysis patients. Although this study achieved satisfactory results, there are still some limitations. First of all, due to the small sample size and enrollment of only hemodialysis patients in a specific region, the results may not be fully generalized to other regions or different populations, necessitating further validation by multi-center, large-sample prospective studies in the future. In addition, although the SF-36 is a well-validated tool for assessing general quality of life, it is not disease-specific. The Kansas City Cardiomyopathy Questionnaire (KCCQ), which is specifically designed for HF patients and captures symptom burden and functional limitations more precisely, may be considered in future studies to enhance the sensitivity and relevance of outcome assessment.

## Data Availability

The original contributions presented in the study are included in the article/Supplementary Material, further inquiries can be directed to the corresponding author.
