# Supplementary material for: Analysis of risk factors and application of risk management strategies in hemodialysis patients complicated with heart failure
Source: Front Cardiovasc Med. 2025 Jun 16;12:1600223. doi: 10.3389/fcvm.2025.1600223 (PMC12206736; doi:10.3389/fcvm.2025.1600223)
Supplement: Supplementary file 1 [file Datasheet1.pdf]

Supplementary Material: Psychological Assessment Scales Used in This Study

(Please fill in the data in parentheses)

This appendix includes the Self-Rating Anxiety Scale (SAS) and the Self-Rating Depression Scale (SDS) used in this study. Each item is scored on a scale from 1 to 4: 1 = None or a little of the time, 2 = Some of the time, 3 = A good part of the time, 4 = Most or all of the time. The raw total score is multiplied by 1.25 to obtain the standard score. A standard score ≥50 indicates a possible presence of anxiety or depression.

Psychological Assessment Scales (SAS)

1. I feel more nervous and anxious than usual.（　）

1：None or a little of the time

2：Some of the time

3：A good part of the time

4：Most or all of the time

2. I felt scared for no reason.（　）

1：None or a little of the time

2：Some of the time

3：A good part of the time

4：Most or all of the time

3. I tend to be annoyed or panicked easily.（　）

1：None or a little of the time

2：Some of the time

3：A good part of the time

4：Most or all of the time

4. I feel that I'm going to break down or lose control of myself.（　）

1：None or a little of the time

2：Some of the time

3：A good part of the time

4：Most or all of the time

5. I feel everything is fine and nothing will go wrong.（　）

1：None or a little of the time

2：Some of the time

3： A good part of the time
4： Most or all of the time

6. My arms and legs are shaking. （ ）
1： None or a little of the time
2： Some of the time
3： A good part of the time
4： Most or all of the time

7. I'm troubled by headache, neck pain and back pain. （ ）
1： None or a little of the time
2： Some of the time
3： A good part of the time
4： Most or all of the time

8. I feel weak and prone to fatigue. （ ）
1： None or a little of the time
2： Some of the time
3： A good part of the time
4： Most or all of the time

9. I can keep calm and sit quietly easily. （ ）
1： None or a little of the time
2： Some of the time
3： A good part of the time
4： Most or all of the time

10. My heart is beating very fast. （ ）
1： None or a little of the time
2： Some of the time
3： A good part of the time
4： Most or all of the time

11. I am troubled by nausea or an uncomfortable stomach. （ ）
1： None or a little of the time
2： Some of the time
3： A good part of the time

4：Most or all of the time

12. I often feel dizzy.（   ）
1：None or a little of the time
2：Some of the time
3：A good part of the time
4：Most or all of the time

13. I have a feeling of chills and fever in waves.（   ）
1：None or a little of the time
2：Some of the time
3：A good part of the time
4：Most or all of the time

14. My hands are often dry and warm.（   ）
1：None or a little of the time
2：Some of the time
3：A good part of the time
4：Most or all of the time

15. My face turned red and hot.（   ）
1：None or a little of the time
2：Some of the time
3：A good part of the time
4：Most or all of the time

16. I often have the feeling of fainting.（   ）
1：None or a little of the time
2：Some of the time
3：A good part of the time
4：Most or all of the time

17. I have difficulty breathing.（   ）
1：None or a little of the time
2：Some of the time
3：A good part of the time
4：Most or all of the time

18. I often have nightmares.（　）
1：None or a little of the time
2：Some of the time
3：A good part of the time
4：Most or all of the time

19. I feel tightness in my chest.（　）
1：None or a little of the time
2：Some of the time
3：A good part of the time
4：Most or all of the time

20. My hands and feet are numb and tingling.（　）
1：None or a little of the time
2：Some of the time
3：A good part of the time
4：Most or all of the time

Self-rating Depression Scale (SDS)

1. I feel depressed and frustrated.（ ）

1：None or a little of the time

2：Some of the time

3：A good part of the time

4：Most or all of the time

2. Morning is the time when I'm in the worst mood.（ ）

1：None or a little of the time

2：Some of the time

3：A good part of the time

4：Most or all of the time

3. I often cry or want to cry.（ ）

1：None or a little of the time

2：Some of the time

3：A good part of the time

4：Most or all of the time

4. I don't sleep well at night.（ ）

1：None or a little of the time

2：Some of the time

3：A good part of the time

4：Most or all of the time

5. I ate as much as usual.（ ）

1：None or a little of the time

2：Some of the time

3：A good part of the time

4：Most or all of the time

6. My intimate relationship with the opposite sex is the same as before.（ ）

1：None or a little of the time

2：Some of the time

3：A good part of the time

4：Most or all of the time

7. I find that my weight is going down.（ ）
1：None or a little of the time
2：Some of the time
3：A good part of the time
4：Most or all of the time

8. I am troubled by constipation.（ ）
1：None or a little of the time
2：Some of the time
3：A good part of the time
4：Most or all of the time

9. My heart beats faster than usual.（ ）
1：None or a little of the time
2：Some of the time
3：A good part of the time
4：Most or all of the time

10. I feel tired for no reason.（ ）
1：None or a little of the time
2：Some of the time
3：A good part of the time
4：Most or all of the time

11. My mind is as clear as usual.（ ）
1：None or a little of the time
2：Some of the time
3：A good part of the time
4：Most or all of the time

12. I do things as easily as usual.（ ）
1：None or a little of the time
2：Some of the time
3：A good part of the time
4：Most or all of the time

13. I was restless and had difficulty keeping calm.（ ）

1：None or a little of the time

2：Some of the time

3：A good part of the time

4：Most or all of the time

14. I have hope for the future.（ ）

1：None or a little of the time

2：Some of the time

3：A good part of the time

4：Most or all of the time

15. I get angry and excited more easily than usual.（ ）

1：None or a little of the time

2：Some of the time

3：A good part of the time

4：Most or all of the time

16. I think it's very easy to make a decision.（ ）

1：None or a little of the time

2：Some of the time

3：A good part of the time

4：Most or all of the time

17. I feel that I am a useful and indispensable person.（ ）

1：None or a little of the time

2：Some of the time

3：A good part of the time

4：Most or all of the time

18. My life is very meaningful.（ ）

1：None or a little of the time

2：Some of the time

3：A good part of the time

4：Most or all of the time

19. I still love the things I usually like.（ ）

1：None or a little of the time
2：Some of the time
3：A good part of the time
4：Most or all of the time

20. I still love the things I usually like.（ ）
1：None or a little of the time
2：Some of the time
3：A good part of the time
4：Most or all of the time
