# Supplementary material for: Analysis of risk factors and application of risk management strategies in hemodialysis patients complicated with heart failure
Source: Front Cardiovasc Med. 2025 Jun 16;12:1600223. doi: 10.3389/fcvm.2025.1600223 (PMC12206736; doi:10.3389/fcvm.2025.1600223)
Supplement: Supplementary file 2 [file Datasheet2.pdf]

Supplementary Material: Simplified SF-36 Scale (Research Version, 20 Items per Dimension) (Please fill in the data in parentheses)

This appendix presents the simplified SF-36 Quality of Life Questionnaire used in this study. It includes five dimensions: physical functioning, social functioning, emotional functioning, mental health, and general health. Each dimension consists of 20 items, with each item scored from 1 to 5 points. The total score for each dimension (ranging from 20 to 100) is calculated by summing the scores of the 20 items. Higher scores indicate better quality of life.

1. Physiological Function（PF）

1. Can one independently complete basic living activities such as washing up and getting dressed?（ ）

1: Very poor 2: Poor 3: Average 4: Good 5: Very good

2. Is it possible to walk more than one kilometer?（ ）

1: Very poor 2: Poor 3: Average 4: Good 5: Very good

3. Is it possible to go up and down the stairs?（ ）

1: Very poor 2: Poor 3: Average 4: Good 5: Very good

4. Can medium-weight items (such as a bucket of water) be lifted?（ ）

1: Very poor 2: Poor 3: Average 4: Good 5: Very good

5. Can household chores (such as sweeping the floor, cooking, etc.) be done?（ ）

1: Very poor 2: Poor 3: Average 4: Good 5: Very good

6. Do you feel tired in daily activities?（ ）

1: Very poor 2: Poor 3: Average 4: Good 5: Very good

7. Is it possible to ride a bike or jog?（ ）

1: Very poor 2: Poor 3: Average 4: Good 5: Very good

8. Is it possible to complete outdoor activities such as shopping?（ ）

1: Very poor 2: Poor 3: Average 4: Good 5: Very good

9. Can one get on and off the bus independently?（ ）

1: Very poor 2: Poor 3: Average 4: Good 5: Very good

10. Has the activity been reduced due to physical reasons? (   )
1: Very poor 2: Poor 3: Average 4: Good 5: Very good

11. Can one walk continuously for 10 minutes without taking a break? (   )
1: Very poor 2: Poor 3: Average 4: Good 5: Very good

12. Can one stand for a long time (more than 30 minutes)? (   )
1: Very poor 2: Poor 3: Average 4: Good 5: Very good

13. Can one take care of daily diet by oneself? (   )
1: Very poor 2: Poor 3: Average 4: Good 5: Very good

14. Have you ever lain in bed to rest due to feeling unwell? (   )
1: Very poor 2: Poor 3: Average 4: Good 5: Very good

15. Is it possible to take part in moderate exercise? (   )
1: Very poor 2: Poor 3: Average 4: Good 5: Very good

16. Do you often stop your activities early due to physical problems? (   )
1: Very poor 2: Poor 3: Average 4: Good 5: Very good

17. Can the toilet be used normally? (   )
1: Very poor 2: Poor 3: Average 4: Good 5: Very good

18. Can I get in and out of bed by myself? (   )
1: Very poor 2: Poor 3: Average 4: Good 5: Very good

19. Is it possible to go out with a backpack? (   )
1: Very poor 2: Poor 3: Average 4: Good 5: Very good

20. Is it possible to adapt to the weather changes and go out? (   )
1: Very poor 2: Poor 3: Average 4: Good 5: Very good

2. Social Function (SF)

21. Do you often communicate with your family? (   )
1: Very poor 2: Poor 3: Average 4: Good 5: Very good

22. Would you like to participate in the friends' gathering? (   )

1: Very poor 2: Poor 3: Average 4: Good 5: Very good

23. Do you often refuse to socialize for health reasons? ( )
1: Very poor 2: Poor 3: Average 4: Good 5: Very good

24. Do you feel that you are part of the social circle? ( )
1: Very poor 2: Poor 3: Average 4: Good 5: Very good

25. Will there be a reduction in contact with others due to health problems? ( )
1: Very poor 2: Poor 3: Average 4: Good 5: Very good

26. Is it possible to maintain contact with close friends? ( )
1: Very poor 2: Poor 3: Average 4: Good 5: Very good

27. Has the frequency of going out for social activities decreased due to illness? ( )
1: Very poor 2: Poor 3: Average 4: Good 5: Very good

28. Would you like to participate in community activities? ( )
1: Very poor 2: Poor 3: Average 4: Good 5: Very good

29. Are you capable of visiting relatives and friends? ( )
1: Very poor 2: Poor 3: Average 4: Good 5: Very good

30. Have you participated in any interest groups or clubs? ( )
1: Very poor 2: Poor 3: Average 4: Good 5: Very good

31. Do you feel a lack of confidence in social interactions? ( )
1: Very poor 2: Poor 3: Average 4: Good 5: Very good

32. Are you worried about sudden health problems when going out? ( )
1: Very poor 2: Poor 3: Average 4: Good 5: Very good

33. Do you feel at ease in public? ( )
1: Very poor 2: Poor 3: Average 4: Good 5: Very good

34. Are you willing to share your life? ( )
1: Very poor 2: Poor 3: Average 4: Good 5: Very good

35. Can one undertake social responsibilities in the family? ( )

1: Very poor   2: Poor   3: Average   4: Good   5: Very good

36. Do you feel that you can be understood and supported? (   )
1: Very poor   2: Poor   3: Average   4: Good   5: Very good

37. Are you interested in social activities? (   )
1: Very poor   2: Poor   3: Average   4: Good   5: Very good

38. Do you feel happy at family gatherings? (   )
1: Very poor   2: Poor   3: Average   4: Good   5: Very good

39. Do you maintain a good relationship with your neighbors? (   )
1: Very poor   2: Poor   3: Average   4: Good   5: Very good

40. Have you avoided socializing due to physical problems? (   )
1: Very poor   2: Poor   3: Average   4: Good   5: Very good

3. Emotional Function (RE)

41. Has the working hours been reduced due to emotional problems? (   )
1: Very poor   2: Poor   3: Average   4: Good   5: Very good

42. Is it easy to give up tasks due to irritability? (   )
1: Very poor   2: Poor   3: Average   4: Good   5: Very good

43. Have you ever been unable to concentrate due to nervousness? (   )
1: Very poor   2: Poor   3: Average   4: Good   5: Very good

44. Are you delaying affairs due to depression? (   )
1: Very poor   2: Poor   3: Average   4: Good   5: Very good

45. Have you ever been in a low mood that affected your decision-making? (   )
1: Very poor   2: Poor   3: Average   4: Good   5: Very good

46. Is there a lack of interest in work/affairs? (   )
1: Very poor   2: Poor   3: Average   4: Good   5: Very good

47. Is it because emotional fluctuations affect communication? (   )
1: Very poor   2: Poor   3: Average   4: Good   5: Very good

48. Do you often get angry over small matters?（ ）
1: Very poor 2: Poor 3: Average 4: Good 5: Very good

49. Has anxiety affected the action plan?（ ）
1: Very poor 2: Poor 3: Average 4: Good 5: Very good

50. Are you easily agitated or lose control of your emotions?（ ）
1: Very poor 2: Poor 3: Average 4: Good 5: Very good

51. Is there any evasion of responsibility due to emotional issues?（ ）
1: Very poor 2: Poor 3: Average 4: Good 5: Very good

52. Do you feel unable to handle your daily roles?（ ）
1: Very poor 2: Poor 3: Average 4: Good 5: Very good

53. Are you lacking confidence in the future?（ ）
1: Very poor 2: Poor 3: Average 4: Good 5: Very good

54. Are you annoyed by trivial matters?（ ）
1: Very poor 2: Poor 3: Average 4: Good 5: Very good

55. Is sleep affected by tension?（ ）
1: Very poor 2: Poor 3: Average 4: Good 5: Very good

56. Are you troubled by unexplained low spirits?（ ）
1: Very poor 2: Poor 3: Average 4: Good 5: Very good

57. Have you ever had an experience where emotions caused chaos in your life?（ ）
1: Very poor 2: Poor 3: Average 4: Good 5: Very good

58. Do you often interrupt your activities due to mental fatigue?（ ）
1: Very poor 2: Poor 3: Average 4: Good 5: Very good

59. Do you have an emotional burden on your family or work?（ ）
1: Very poor 2: Poor 3: Average 4: Good 5: Very good

60. Have you ever sought help for emotional problems?（ ）
1: Very poor 2: Poor 3: Average 4: Good 5: Very good

4. Mental Health (MH)

61. Do you feel emotionally stable?（　）
1: Very poor　2: Poor　3: Average　4: Good　5: Very good

62. Do you often feel happy?（　）
1: Very poor　2: Poor　3: Average　4: Good　5: Very good

63. Are you full of hope for life?（　）
1: Very poor　2: Poor　3: Average　4: Good　5: Very good

64. Do you feel anxious or uneasy?（　）
1: Very poor　2: Poor　3: Average　4: Good　5: Very good

65. Can one handle the stress in life?（　）
1: Very poor　2: Poor　3: Average　4: Good　5: Very good

66. Is it easy to be pessimistic or worried about the future?（　）
1: Very poor　2: Poor　3: Average　4: Good　5: Very good

67. Do you feel that life is meaningful?（　）
1: Very poor　2: Poor　3: Average　4: Good　5: Very good

68. Is it easy to control emotional fluctuations?（　）
1: Very poor　2: Poor　3: Average　4: Good　5: Very good

69. Can you feel the care around you?（　）
1: Very poor　2: Poor　3: Average　4: Good　5: Very good

70. Do you often feel lonely?（　）
1: Very poor　2: Poor　3: Average　4: Good　5: Very good

71. Can one happily engage in hobbies and interests?（　）
1: Very poor　2: Poor　3: Average　4: Good　5: Very good

72. Is there a sufficient emotional support system?（　）
1: Very poor　2: Poor　3: Average　4: Good　5: Very good

73. Can one handle complex interpersonal relationships?（　）
1: Very poor 2: Poor 3: Average 4: Good 5: Very good

74. Do you feel that your daily rhythm is under control?（　）
1: Very poor 2: Poor 3: Average 4: Good 5: Very good

75. Do you feel mentally pleasant?（　）
1: Very poor 2: Poor 3: Average 4: Good 5: Very good

76. Do you maintain motivation for work/life?（　）
1: Very poor 2: Poor 3: Average 4: Good 5: Very good

77. Are you often disappointed in yourself?（　）
1: Very poor 2: Poor 3: Average 4: Good 5: Very good

78. Are you evading reality due to mental distress?（　）
1: Very poor 2: Poor 3: Average 4: Good 5: Very good

79. Is the diet affected by psychological problems?（　）
1: Very poor 2: Poor 3: Average 4: Good 5: Very good

80. Do you often have headaches or insomnia due to stress?（　）
1: Very poor 2: Poor 3: Average 4: Good 5: Very good

5. General Health (GH)

81. How do you evaluate your health in general?（　）
1: Very poor 2: Poor 3: Average 4: Good 5: Very good

82. Do you think your health condition is good now?（　）
1: Very poor 2: Poor 3: Average 4: Good 5: Very good

83. Are you prone to catching a cold or getting sick?（　）
1: Very poor 2: Poor 3: Average 4: Good 5: Very good

84. Has your health improved compared to the past year?（　）
1: Very poor 2: Poor 3: Average 4: Good 5: Very good

85. Are you worried about the development of chronic diseases?（　）

1: Very poor 2: Poor 3: Average 4: Good 5: Very good

86. Are you confident about your future health? （ ）
1: Very poor 2: Poor 3: Average 4: Good 5: Very good

87. Do you have regular physical examinations? （ ）
1: Very poor 2: Poor 3: Average 4: Good 5: Very good

88. Do you control your diet to stay healthy? （ ）
1: Very poor 2: Poor 3: Average 4: Good 5: Very good

89. o you exercise regularly? （ ）
1: Very poor 2: Poor 3: Average 4: Good 5: Very good

90. Do you feel that your physical condition is affecting your life? （ ）
1: Very poor 2: Poor 3: Average 4: Good 5: Very good

91. Do you think you are healthier than your peers? （ ）
1: Very poor 2: Poor 3: Average 4: Good 5: Very good

92. Are you troubled by your current illness? （ ）
1: Very poor 2: Poor 3: Average 4: Good 5: Very good

93. Do you need to take medicine frequently? （ ）
1: Very poor 2: Poor 3: Average 4: Good 5: Very good

94. Are you satisfied with the current treatment? （ ）
1: Very poor 2: Poor 3: Average 4: Good 5: Very good

95. Do you often need to be hospitalized or see a doctor due to illness? （ ）
1: Very poor 2: Poor 3: Average 4: Good 5: Very good

96. Do you attach importance to health maintenance in your daily life? （ ）
1: Very poor 2: Poor 3: Average 4: Good 5: Very good

97. Do you think your health condition limits your activities? （ ）
1: Very poor 2: Poor 3: Average 4: Good 5: Very good

98. Do you often feel tired and weak? （ ）

1: Very poor   2: Poor   3: Average   4: Good   5: Very good

99. Have your social life been affected by health problems?（　）
1: Very poor   2: Poor   3: Average   4: Good   5: Very good

100. Would you like to change your lifestyle to improve your health?（　）
1: Very poor   2: Poor   3: Average   4: Good   5: Very good
