# Supplementary figures and images for: Analysis of risk factors and application of risk management strategies in hemodialysis patients complicated with heart failure
Source: Front Cardiovasc Med. 2025 Jun 16;12:1600223. doi: 10.3389/fcvm.2025.1600223 (PMC12206736; doi:10.3389/fcvm.2025.1600223)

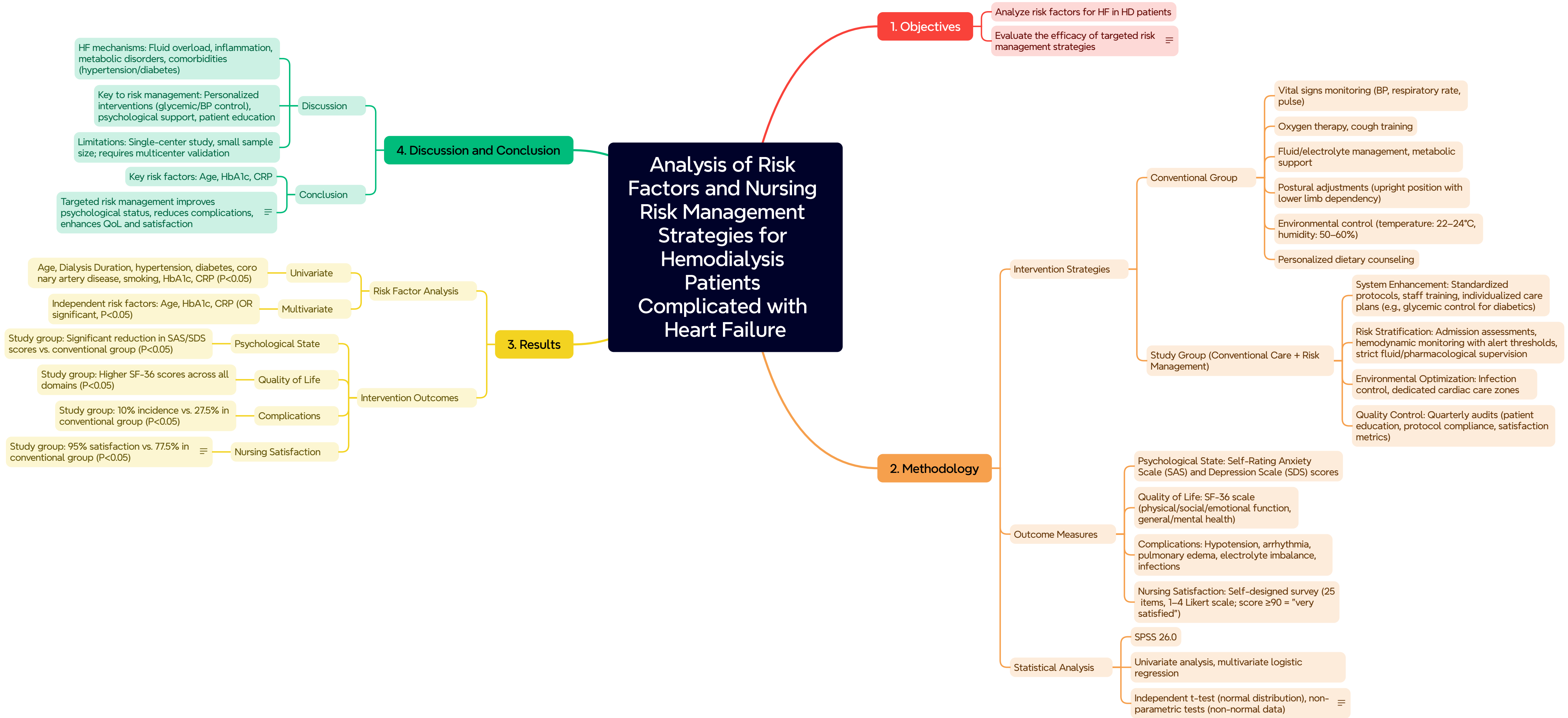

Supplement: Supplementary file 3 [file Image1.pdf]
